# Impact of Moderate to Severe Renal Impairment on Mortality and Appropriate Shocks in Patients with Implantable Cardioverter Defibrillators

**DOI:** 10.4061/2010/150285

**Published:** 2010-12-15

**Authors:** Venkata M. Alla, Kishlay Anand, Mandeep Hundal, Aimin Chen, Showri Karnam, Tom Hee, Claire Hunter, Aryan N. Mooss, Dennis Esterbrooks, Syed M. Mohiuddin

**Affiliations:** ^1^Division of Cardiology, Creighton University Medical Center, 3006 Webster Street, Omaha, NE 68131, USA; ^2^Department of Cardiology, University of California, San Diego, CA 92103, USA; ^3^Department of Internal Medicine, Cheyenne Regional Medical Center, Cheyenne, WY 82001, USA; ^4^Department of Internal Medicine, Creighton University Medical Center, Omaha, NE 68131, USA

## Abstract

*Background*. Due to underrepresentation of patients with chronic kidney disease (CKD) in large Implantable-Cardioverter Defibrillator (ICD) clinical trials, the impact of ICD remains uncertain in this population. *Methods*. Consecutive patients who received ICD at Creighton university medical center between years 2000–2004 were included in a retrospective cohort after excluding those on maintenance dialysis. Based on baseline Glomerular filtration rate (GFR), patients were classified as severe CKD: GFR < 30 mL/min; moderate CKD: GFR: 30–59 mL/min; and mild or no CKD: GFR ≥ 60 mL/min. The impact of GFR on appropriate shocks and survival was assessed using Kaplan-Meier method and Generalized Linear Models (GLM) with log-link function. *Results*. There were 509 patients with a mean follow-up of 3.0 + 1.3 years. Mortality risk was inversely proportional to the estimated GFR: 2 fold higher risk with GFR between 30–59 mL/min and 5 fold higher risk with GFR < 30 mL/min. One hundred and seventy-seven patients received appropriate shock(s); appropriate shock-free survival was lower in patients with severe CKD (GFR < 30) compared to mild or no CKD group (2.8 versus 4.2 yrs). *Conclusion*. Even moderate renal dysfunction increases all cause mortality in CKD patients with ICD. Severe but not moderate CKD is an independent predictor for time to first appropriate shock.

## 1. Introduction

Chronic kidney disease (CKD) is present among 17% of the US adult population and is a strong and independent predictor of cardiovascular events, ventricular arrhythmias, and sudden death [1, 2]. This is true even in early stages of CKD and in patients who are not dialysis dependant [2]. Sudden cardiac death (SCD) accounts for up to 25% of the all cause mortality and 60% of cardiac deaths in dialysis-dependant patients [1, 3]. Multiple trials have demonstrated the survival benefits of Implantable-Cardioverter Defibrillators (ICD) in the primary and secondary prevention of SCD in patients fulfilling broad inclusion criteria [4, 5]. However, due to underrepresentation of patients with CKD in these large ICD clinical trials, outcomes remain uncertain in this population. Prior observational studies have shown that despite the high risk of SCD, patients with advanced CKD do not derive significant benefit from ICD implantation and continue to have substantial mortality [6–10]. However, a majority of the above trials included patients with end-stage renal failure on maintenance dialysis. The impact of less advanced CKD (non dialysis dependant) in patients with ICD remains unclear. Similarly, prior studies have shown that need for dialysis is a predictor of appropriate ICD therapy; however, the impact of moderate CKD on the frequency and time to appropriate shock is less explored.

## 2. Methods

 This was a retrospective cohort study and included consecutive patients who underwent ICD implantation at Creighton University Medical Center from January 2000 to December 2004. Patients with end-stage renal failure on maintenance dialysis at the time of implantation were excluded. Baseline demographics, clinical, echocardiographic, laboratory, and treatment data including indication for ICD implantation (primary or secondary), New York Heart Association (NYHA) class of heart failure, Left Ventricular Ejection Fraction (LVEF), QRS duration, and medications were noted. All patients undergoing device implantation in our institute get a basic metabolic panel and complete blood count as a part of preoperative work up within a week before surgery. Thus, baseline renal function data within 7 days preceding surgery was available in all patients. During followup, the time from ICD implantation to the first appropriate ICD shock was ascertained by reviewing device interrogation and clinic records. Appropriate shock was defined as a shock delivered secondary to ventricular tachycardia or ventricular fibrillation as per treating Electrophysiologist's interpretation (shocks for supra ventricular arrhythmias and those due to device malfunction were excluded). The survival status was ascertained from review of medical records and verified from online Social Security Death Index database. We used the baseline variables to estimate GFR using the simplified Modification of Diet in Renal Disease (sMDRD) study prediction equation [11]. 

The sMDRD (mL/min per 1.73 m^2^) is calculated as follows:

- Male: 186.3 × (serum  creatinine)^−1.154^ × (age)^−0.203^;
- Black male: sMDRD × 1.212;
- Female: sMDRD × 0.742;
- Black female: sMDRD × 1.212 × 0.742.

Patients were stratified into 3 groups: normal renal function or mild CKD, moderate CKD, and severe CKD. Based on the National kidney Foundation classification, CKD stage 1 and 2 constituted group 1, CKD stage 3 constituted group 2, and CKD stages 4-5 constituted group 3 [12]. The objective of this study was to determine the effect of moderate to severe CKD not requiring maintenance dialysis on the time to first appropriate ICD shock and all-cause mortality. The study was approved by the Institutional Review Board at Creighton University.

## 3. Statistical Analysis

 The baseline characteristics of the groups were compared using student *t*-test for continuous variables and the *χ* ^2^ statistic for categorical variables. Continuous variables were reported as mean with standard deviation and categorical variables were reported as percentage. Survival analysis for time to first ICD shock was analyzed using Kaplan-Meier method. Univariate and multivariate Cox proportional hazard models were fitted to derive unadjusted and adjusted hazards for time to first appropriate shock for variables of interest. Predictors of mortality were assessed using Generalized Linear Models (GLMs) with log-link function to account for high risk of death in the study sample; risk ratios (RRs) and 95% confidence intervals (CI) were calculated. All statistical analyses were performed using SAS 9.1 (SAS Institute Inc, Cary, NC).

## 4. Results

### 4.1. Baseline Characteristics

 Five hundred and nine consecutive patients with a mean followup of 3.0 years (Standard Deviation [SD] 1.3 years) were included in the study. Followup data was not available in 27 patients.  Table 1 shows the characteristics of patients with ICD stratified by GFR. Patients with lower GFR were older and more often had ischemic heart disease as the underlying etiology. In addition, they had a worse functional status (NYHA class ≥3) and a tendency towards lower hemoglobin. Medication use including beta-blockers, angiotensin converting enzyme (ace) inhibitors and antiarrhythmics was similar between the 2 groups except for loop diuretics which were used more often in the patients with lower GFR. 

### 4.2. Time to First Appropriate Shock

**Figure 1:**
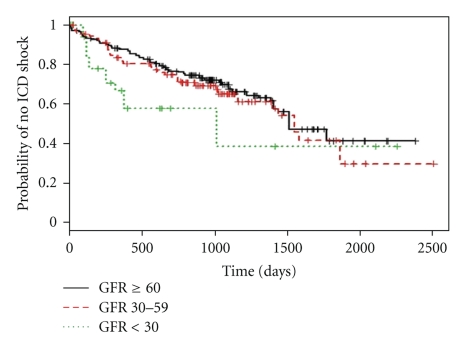
Kaplan-Meier curves for time to first appropriate ICD shock based on GFR. The “+” sign in the graph indicates censoring.

 One hundred seventy seven patients (35%) received appropriate shock during a mean followup of 3 years. Kaplan Meier curves showing shock free survival for the 3 GFR groups is represented in Figure 1. Patients with moderate CKD had similar median shock-free survival time as patients with mild or no CKD. However, significantly shorter median shock-free survival time was noted in patients with severe CKD (GFR < 30 mL/min) compared to those with GFR ≥ 60 mL/min (2.8 versus 4.2 yrs). The hazard ratios (HR) from Cox proportional hazard models for time to first appropriate shock by CKD stages are shown in Table 2. In the multivariate Cox proportionate hazard model, LVEF < 30% and admission for heart failure were also independently associated with shorter time to shock. On the other hand, ICD implantation for primary prevention was associated with longer time to shock. Age, NYHA functional class, beta-blockers, and antiarrhythmic drug use were not independently associated with time to first appropriate shock. Though information on antitachycardia pacing (ATP) was recorded wherever available, the data pertaining to appropriate ATP was missing in many patients. Furthermore, ICD programming pertaining to number of zones (VT, VF) and ATP programming was extremely variable during the study period. Hence, we did not analyze time to appropriate ATP in this study.

### 4.3. Mortality

**Figure 2:**
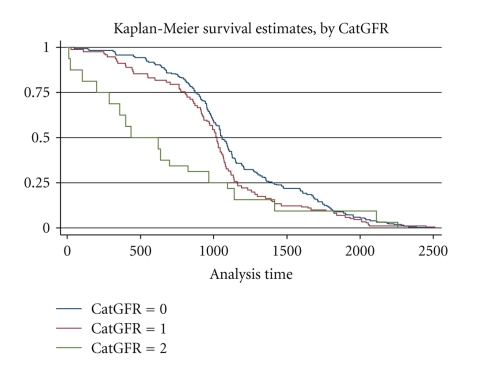
Kaplan-Meier curves for survival based on GFR. CatGFR: 0 implies GFR ≥ 60; catGFR: 1 implies GFR 30–59; catGFR: 2 implies GFR < 30 mL/min.

 The risk of death in ICD patients stratified based on renal function is shown in Table 3. Moderate CKD (GFR 30–59 mL/min) had approximately twofold increased risk of all-cause mortality compared with mild or no CKD (GFR > 60 mL/min). Severe CKD (GFR < 30 mL/min) had about fivefold higher risk of all-cause mortality. Kaplan Meier curves for survival based on GFR are shown in Figure 2. In the multiple GLM models, LVEF < 30%, increased age, worse NYHA class, no betablocker, use of aldosterone inhibitor or digoxin and admission for heart failure were predictive of increased mortality. The relationship between renal function and mortality remained unchanged when serum creatinine was used in place of derived GFR (Table 4). The risk of death increased progressively from 1st to 4th quartile of serum creatinine (*P* for trend <  .01). 

## 5. Discussion

The principal finding of our study is that even moderate degree of renal impairment is associated with increased all-cause mortality in patients with ICD. Moreover, there was a graded response with lower GFR conferring a proportionately higher risk: GFR < 30 mL/min had fivefold higher risk of all-cause mortality and GFR 30–59 mL/min had twofold higher risk of all-cause mortality. Additionally, we demonstrated that CKD is associated with increased risk for appropriate shock. However, with respect to ICD shocks, only patients with GFR < 30 mL/min had a significantly shorter implantation to ICD shock time. Though patients with moderate CKD (GFR 30–59 mL/min) had a trend towards earlier shock, this did not reach statistical significance. The findings of our study are in line with previous studies on the impact of CKD in patients with ICD [6–10]. In contrast to prior studies, we excluded patients with end-stage renal failure on dialysis in our study. 

 The high rate of arrhythmias in patients with CKD has been attributed to several pathophysiologic mechanisms including diastolic dysfunction, cardiac interstitial fibrosis, autonomic dysfunction, and adverse pharmacologic interactions [13–16]. Potential reasons for high mortality in CKD patients despite ICD implantation could be the limited efficacy of ICD in preventing arrhythmic death due to inappropriately high defibrillation thresholds (DFT) or non-SCD mechanisms for mortality [17–19]. Prior reports have shown that DFT increases as a function of renal insufficiency [7]. Alternatively, mortality from competing risk factors could minimize the benefits of ICD. The most commonly reported underlying cause of death in patients with ICD and CKD is progressive heart failure [10]. In the study by Eckart et al., hospital admission for heart failure was three times more common in ICD patients with CKD compared to those without renal insufficiency [17]. Infection and vascular events are other common reasons for death in patients with advanced renal failure, and could explain why the survival benefits of ICD are attenuated in this population [18, 19]. In our study advanced age, poor NYHA class, lower LVEF, use of digoxin and aldosterone inhibitors were other predictors of all-cause mortality. Beta-blocker had a significant protective effect on all-cause mortality and statins (hydroxy-methyl-glutaryl coenzyme-A inhibitors) showed a trend towards decreased mortality. In contrast to prior reports, beta-blockers only demonstrated a trend towards decreasing ICD discharge that did not reach statistical significance in our study [20]. Aldosterone inhibitors have proven benefits in patients with heart failure and low LVEF [21]; however, these studies excluded patients with moderate to severe CKD. The adverse mortality effects of digoxin and aldosterone inhibitors may be due to combination of poor baseline status (reflective of more advanced heart disease) or increased adverse effects of these drugs in CKD populations (like hyperkalemia). 

In a retrospective analysis of the patients enrolled in the Multicenter Automatic Defibrillator Implantation Trial-II, ICD implantation significantly reduced mortality in patients with estimated GFR > 35 ml/min/1.73 m^2^ (risk reduction for all-cause mortality 32%, *P* = .01), but had no benefit in patients with a GFR < 35 ml/min/1.73 m^2^ (all-cause mortality hazard ratio 1.09, *P* = .84) [22]. On the other hand, Herzog et al. demonstrated survival benefit with ICD implantation in dialysis-dependant patients following aborted cardiac arrest [23]. Notably, the limited efficacy of therapy in patients with renal dysfunction is not unique to ICD. There is evidence suggesting that the benefits of other life saving interventions like coronary angioplasty and coronary artery bypass grafting are similarly blunted in patients with CKD compared to those without [3, 24]. Thus, in patients who are candidates for ICD, the presence of renal impairment creates a unique and complex set of issues. The incidence of cardiovascular events, ventricular arrhythmias, and sudden death is high indicating high likelihood of benefit. On the other hand, the beneficial effects of ICD appear to be less than expected or in fact questionable. This is likely to be multifactorial, related to the diminished efficacy of ICDs in these patients, higher rates of device-related complications like infection and competing risks like heart failure, and vascular disease which increase nonarrhythmic mortality [17–19, 24–27]. In our study, 33 patients (7%) developed device-related complications (early and late) which included pocket infection, hematoma needing exploration, pneumothorax, endocarditis, and cardiac perforation by ICD lead. There was no significant difference among the different GFR groups. 

 Additionally, there is evidence to suggest that ICDs like all other life saving medical and surgical interventions are grossly underutilized in CKD populations; less than 10% of renal failure patients surviving a sudden cardiac arrest get an ICD [3, 23]. While “therapeutic nihilism” is one potential contributing factor, the lack of strong evidence base supporting the benefit of these interventions is another major limitation. Given the controversies about potential overestimation of ICD benefits (at least in the primary prevention setting) and the concerns about cost-effectiveness, it is imperative for researchers to evaluate the benefits of ICDs in CKD populations in well-designed large scale prospective randomized trials [27]. Furthermore, risk prediction models that differentiate between CKD patients who will and will not benefit from ICD implantation need to be developed and prospectively validated to ensure optimal selection of candidates. In a provocative paper, Amin et al. determined that the benefit of ICD in patients with renal insufficiency is limited to patients with age <80 for stage 3, age <75 for stage 4, and age <65 for stage 5 using a complex prediction model [28].

## 6. Strengths and Limitations

 Our study is a single center, retrospective observational study. As is the case with all observational studies, the quality of data is limited by accuracy of data collection and storage. We made an exhaustive effort to collect all data elements and multiple record sources with linking techniques being used in order to minimize errors and deficiencies. We were however, unable to ascertain the cause of death. In addition, creatinine clearance was estimated based on a one time measurement; hence, there is a potential for misclassification bias. Similarly, residual confounding cannot be excluded despite statistical adjustments for known confounders due to the retrospective nature of the study. An additional limitation is that the study population was predominantly Caucasian and males; hence it was not a representative sample. However, this is reflective of real word ICD implantation practices as it is well known that female sex and African American race are associated with lower rates of ICD implantation [29, 30]. Furthermore, majority of our patients had ischemic heart disease as the underlying substrate. An additional limitation was the use of time to shock rather than time to appropriate ICD therapy (shock and ATP). Though the latter is a more comprehensive end point, ICD shock has more clinical relevance. Moreover, ATP programming is extremely variable and determining if it is appropriate versus inappropriate is more prone for error. The relatively large number of patients studied is a major strength of our study. Only a couple of studies of similar size have assessed the association between CKD and mortality in ICD populations [6, 10, 17, 31]. Unlike other studies, our study excluded dialysis-dependant patients who have a very high mortality. Despite this, we could demonstrate a strong association between moderate-severe CKD and mortality. In addition, our study evaluated the time to appropriate shock which has not previously been studied in patients with moderate-severe CKD.

## 7. Conclusions

 Our results support findings from previous studies showing higher mortality in ICD patients with CKD [6–10, 17, 31]. This is true even with moderate degree of renal dysfunction and nondialysis-dependant patients. In addition, our study demonstrates that patients with severe CKD have lower shock-free survival. There is an urgent need to develop and test risk scoring systems incorporating GFR along with other clinical factors that can reliably identify CKD patients who will benefit from ICD implantation. The need for large, randomized, and prospective clinical trials assessing the impact of ICD on survival, quality of life, and cost-effectiveness in patients with CKD cannot be overstated.

## Figures and Tables

**Table 1:** Baseline characteristics of the study population.

	GFR ≥ 60	GFR 30–59	GFR < 30	*P*-value
	(*n* = 289)	(*n* = 188)	(*n* = 32)	
Age (years)	68	69	73	.04
Male Gender (%)	92	88	83	.10
DM	32	36	40	.08
Hypertension	71	78	82	.9
NYHA class ≥ 3 (%)	51	63	71	.04
QRS > 120 msec (%)	30	49	54	.40
Ejection Fraction (mean)	32	32	28	.60
Ischemic heart disease	77	83	89	.04
BUN (mean)	20	34	48	<.03
Creatinine (mean)	1.0	1.4	1.8	<.01
Hemoglobin (mean)	14.0	13	12.6	.06
Indication:Primary Prevention (%)	72	76	84	.08
*β* blocker (%)	74	78	78	.97
ACEi (%)	63	58	54	.28
ARB (%)	14	22	21	.28
Aldosterone inhibitor (%)	6	8	9	.39
Digoxin (%)	52	50	51	.85
Loop diuretic (%)	48	64	68	.03
Statin (%)	69	64	60	.29
Anti-arrhythmic (%)	19	28	32	.44

DM: diabetes mellitus; BUN: blood urea Nitrogen; ACEi: angiotensin converting enzyme inhibitors; ARB: angiotensin receptor blockers; NYHA: New York heart association; Statin: hydroxyl-methyl-glutaryl CoA inhibitors.

**Table 2:** Hazard ratios with 95% confidence intervals for time to first appropriate shock based on GFR.

	Unadjusted	Adjusted*
GFR (mL/min/1.73 m^2^)		
≥60	ref	ref
30–59 (moderate CKD)	1.15 (0.84–1.58)	1.15 (0.83–1.58)
<30 (severe CKD)	1.92 (1.09–3.38)	2.42 (1.35–4.34)

*Adjusted for age (≥75 or <75 years), sex, indication for ICD implantation (primary/secondary), NYHA class, heart failure admission, antiarrhythmic/betablocker use, and LVEF (<30%, ≥30%).

**Table 3:** Risk ratios and 95% confidence intervals for all-cause mortality based on GFR.

GFR (mL/min/1.73 m^2^)	*n* ^*¶*^	Death (%)	Unadjusted	Adjusted*
≥60	278	11.9	ref	ref
30–59	172	22.5	1.89 (1.24–2.90)	1.81 (1.18–2.78)
<30	32	57.1	4.81 (3.13–7.40)	4.63 (3.02–7.09)

*Adjusted for age (≥75 or <75 years), sex, indication for ICD implantation (primary/secondary), NYHA class, antiarrhythmics, ace inhibitor, digoxin, aldosterone blocker, and betablocker use and LVEF (<30%, ≥30%).

^*¶*^Followup data not available in 27 patients.

**Table 4:** Risk ratios and 95% confidence intervals for all-cause mortality based on serum creatinine.

Serum creatinine (mg/dL)	*n*	Death (%)	Unadjusted	Adjusted*
1st quartile (<1.0)	108	7.41	ref	ref
2nd quartile (1.0–)	128	13.28	1.79 (0.81–3.99)	1.77 (0.80–3.94)
3rd quartile (1.2–)	110	20.00	2.70 (1.26–5.80)	2.72 (1.28–5.82)
4th quartile (1.4–)	136	32.35	4.37 (2.15–8.88)	3.73 (1.84–7.58)

*Adjusted for age (≥75 or <75 years), sex, indication for ICD implantation (primary, secondary), NYHA class, antiarrhythmics, ace inhibitor, digoxin, aldosterone blocker, and betablocker use and LVEF (<30%, ≥30%).
